# A New Approach Using Targeted Sequence Capture for Phylogenomic Studies across Cactaceae

**DOI:** 10.3390/genes13020350

**Published:** 2022-02-15

**Authors:** Serena Acha, Lucas C. Majure

**Affiliations:** Florida Museum of Natural History, University of Florida, Gainesville, FL 32611, USA; llmajure@floridamuseum.ufl.edu

**Keywords:** cacti, Opuntia, Hyb-seq, neotropics, target enrichment

## Abstract

Relationships within the major clades of Cactaceae are relatively well known based on DNA sequence data mostly from the chloroplast genome. Nevertheless, some nodes along the backbone of the phylogeny, and especially generic and species-level relationships, remain poorly resolved and are in need of more informative genetic markers. In this study, we propose a new approach to solve the relationships within Cactaceae, applying a targeted sequence capture pipeline. We designed a custom probe set for Cactaceae using MarkerMiner and complemented it with the Angiosperms353 probe set. We then tested both probe sets against 36 different transcriptomes using Hybpiper preferentially retaining phylogenetically informative loci and reconstructed the relationships using RAxML-NG and Astral. Finally, we tested each probe set through sequencing 96 accessions, representing 88 species across Cactaceae. Our preliminary analyses recovered a well-supported phylogeny across Cactaceae with a near identical topology among major clade relationships as that recovered with plastome data. As expected, however, we found incongruences in relationships when comparing our nuclear probe set results to plastome datasets, especially at the generic level. Our results reveal great potential for the combination of Cactaceae-specific and Angiosperm353 probe set application to improve phylogenetic resolution for Cactaceae and for other studies.

## 1. Introduction

The Cactaceae are a major American radiation consisting of more than 1800 species [1], and they range from Patagonia to Canada [2] in a diversity of habitat types from desert to seasonally dry tropical forests, temperate forests and montane Andean grasslands. Phylogenetic research over the past nearly 30 years has transformed our knowledge of the evolutionary history of the clade. However, most phylogenetic reconstructions have been based solely or mostly on plastid data derived from Sanger sequencing methods and oftentimes using just a handful of loci (e.g., [3,4,5,6,7,8,9]). Arakaki et al. [10] employed a combination of plastome and Sanger sequencing data to test the diversification of the family, and more recently transcriptome data (Walker et al. 2018), as well as plastome datasets [11,12,13,14,15] have been utilized to more fully resolve species limits and deep phylogenetic history in the family. However, nuclear probe sets derived from single-copy loci, which are commonly used in other groups of Angiosperms [16], as well as other vascular and non-vascular plants [17], have not yet been generated for the family. The Angiosperms353 probe set has been widely used to study the relationships in different groups, such as Commelinales [18], Myrtales [19] and Cornales [20]. Additionally, Angiosperms353 has also been combined with group specific probe sets, such as in the case of the Malinae (Rosaceae) [21], Asteraceae [22], Gesneriaceae [23] and Ochnaceae [24]. Finally, there have also been approaches where group-specific probe sets were designed to study different groups of plants at different scales, such as in the genus *Burmeistera* (Campanulaceae) [25], Annonaceae [26] and Zingiberales [27].

In this paper, we describe a 120 locus, single-copy nuclear probe set (aka, Cactaceae120) generated from transcriptome data. We also include a subset of the Angiosperms353 to contribute to the knowledge of Cactaceae relationships within Angiosperms. We explore the gene recovery and phylogenetic reconstructions of both probe sets with 36 publicly available transcriptomes. Additionally, we report the gene recovery results for 96 sequenced samples across all major clades of Cactaceae.

## 2. Materials and Methods

We applied a similar methodology to Jantzen et al. [28]; however, we used 36 transcriptomes (Table 1) instead of genome-skimming sequences to test our locus set performance. We first designed a Cactaceae-specific probe set (from now on referred to as Cactaceae120 or C120). Additionally, we included a subset of the Angiosperms353 (A353) probe set [16], which was shown to have coverage across Caryophyllales, and thus was potentially applicable to Cactaceae. We describe in the following sections the details of both probe sets.

### 2.1. Probe Sets

#### 2.1.1. Cactaceae120

**Figure 1:**
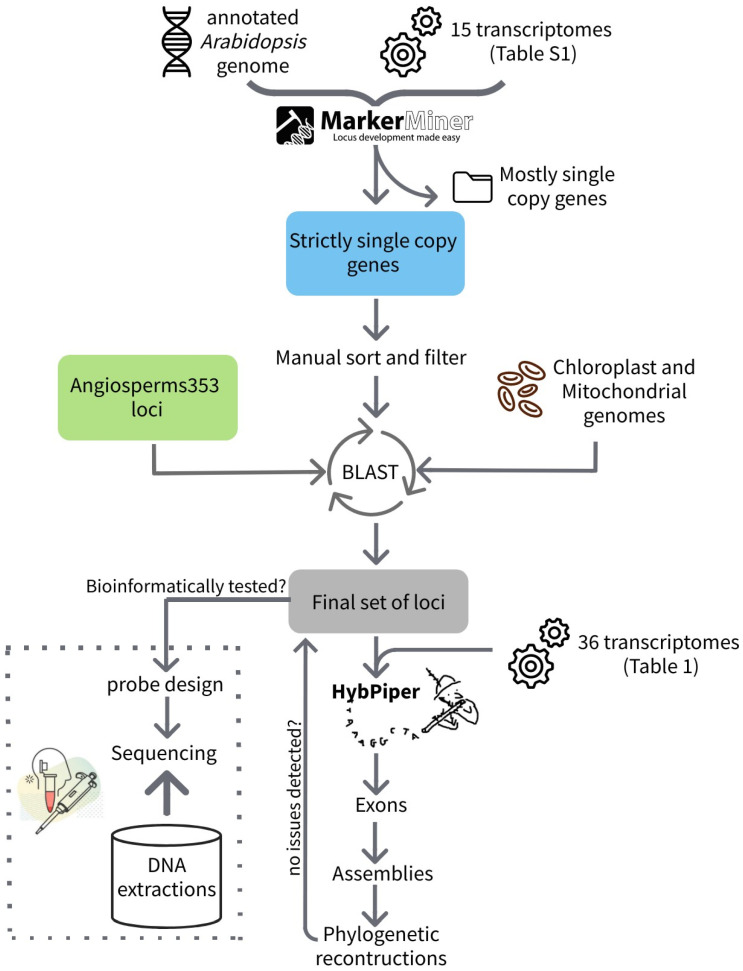
Flowchart of the pipeline with general steps and tools. For more details see the Methods Section.

For the purpose of this study, we used MarkerMiner 1.0 (MM) [29], as implemented in the University of Florida High-Performance Cluster (Hipergator) (Figure 1). We used 15 transcriptomes representing all the main clades in Cactaceae (Table S1). In addition, we selected the *Arabidopsis thaliana* reference genome (TAIR10, [30]) as the closest relative to Cactaceae among the datasets implemented in MarkerMiner. We then inspected the results from MarkerMiner and focused exclusively on the strictly single-copy loci. We manually trimmed our locus sequences in Geneious Prime 2020.0.5 (https://www.geneious.com accessed on 1 August 2020) to include only single-copy loci that contained at least one suitable exon of >120 bp size and intronic regions of 100 bp or more. To avoid the inclusion of any non-nuclear loci, we performed several BLASTx analyses among all the target sequences and (1) *Beta* *vulgaris* and *A. thaliana* mitochondrial genomes, and (2) *A. thaliana* and *Cylindropuntia bigelovii* chloroplast genomes [11,31]. In addition, we performed a BLASTx analysis with the *A. thaliana* whole nuclear genome (Araport11, [32]) to confirm and update the MM annotation results. At the same time, we explored each single-copy locus potential annotations from the GenBank and TAIR database. Finally, we reduced to one copy any identical loci after a reciprocal BLAST between the MarkerMiner, Angiosperms353, and within each probe set.

#### 2.1.2. Angiosperms353

We used the subset of the sequences for Caryophyllales that were recovered for *Nepenthes mirabilis*. This subset included the 296 genes reported for *Nephentes* plus 26 additional genes retrieved from other Caryophyllales accessions in Johnson et al. [16] for a total of 322 genes (SM1 and Table S2).

### 2.2. Bioinformatic Evaluation

We downloaded 36 transcriptomes (Table 1) and examined them using secapr 2.1.1 [33]. We first checked the quality of the sequences and the absence of barcodes with fastqc. We then proceeded to use the Hybpiper 1.3.1 pipeline for both probe sets [34]. We used the default options for nucleotide analysis with the exception that we chose the the Burrows–Wheeler alignment method [35] to search the transcriptome alignments for hits to our target sequences. We then inspected our results with a heatmap plot generated in R using a script by [34] and the R packages ggplot2 and reshape2 [36,37]. Finally, we assessed paralogy using the method implemented in Hybpiper: Mafft and FastTree [34].

### 2.3. Phylogenetic Reconstruction

As a complement to our Hybpiper results and to assess phylogenetic informativeness of both probe sets, we reconstructed the phylogenetic relationships between the 36 transcriptome accessions and compared them to the most widely recovered Cactaceae relationships (e.g., [6,38,39]; reviewed in [40,41]). For this purpose, we extracted the exonic regions of each probe set from the transcriptomes with Hybpiper. We then aligned each locus assembly using MAFFT v7.294b with a gap open penalty of 3 and a gap extension penalty of 0.123 [42]. Finally, we proceeded to clean the MAFFT alignments with trimAI 1.2 [43], allowing only 10% or fewer gaps in the sequences.

Once we cleaned the assemblies for all our loci, we built several data matrices based on the type of analyses needed: (1) two different assemblies were built by concatenating C120 and A353 results separately with annotated partitions based on each locus dataset size; (2) a combined concatenated dataset that included both C120 and A353 with partitions annotations, and (3) the unmodified results from trimAI grouped per probe set and a global combined set (C120 + A353). For all the concatenated datasets, we ran RAxML 8.2.10 [44] with the GTRCAT model with a multiple bootstrap analysis of 500 alternative runs. We then proceeded to build gene trees for all locus assemblies using the Pargenes pipeline [45]. Pargenes implements parallel model selection with modeltest-ng [46], phylogenetic reconstruction on RAxML-NG [47], and the coalescent-based species ASTRAL III [48] approach. Finally, we summarized the gene tree discordance with phyparts [49] and pie charts based on Matt Johnson notebook and scripts (https://github.com/mossmatters/MJPythonNotebooks/blob/master/PhyParts_PieCharts.ipynb accessed on 1 March 2021).

### 2.4. Experimental Evaluation

Once we had the final set of loci that included C120 and A353, we proceeded to test both sets experimentally to prove their efficacy in a diverse set of species (Table S6). We extracted DNA from silica gel dried samples in the Majure Lab at the University of Florida Herbarium (FLAS). We used a modified CTAB buffer-silica column cleaning DNA extraction protocol [11,50]. We then quantified the DNA concentration using a Qubit Fluorometer (Invitrogen) and sent the DNA samples to Rapid Genomics for library preparation and sequencing. Rapid Genomics designed the library of probes based on our reference target sequences from the C120 loci set and the A353 subset for Caryophyllales. All possible probes (120 nt—tiled3x) were designed in silico on a set of 294 loci (RG_3929) with start–end coordinates provided as target subsequences. From the total of all possible probes within the candidate locus sequences, filters were applied to select a set of 4515 probes that were used for hybridization. Removed probes with homopolymers and probes with a similarity of 98% identity for at least 80 bp of the probe length between probes were collapsed. The sequencing was performed on an Illumina HiSeq X platform using paired-end (150 bp) reads. These sequences are the primary dataset of a current research project (Acha and Majure, in prep.); we therefore only report the exploratory results of Hybpiper in this paper.

## 3. Results

### 3.1. Cactaceae120 Loci

**Figure 2:**
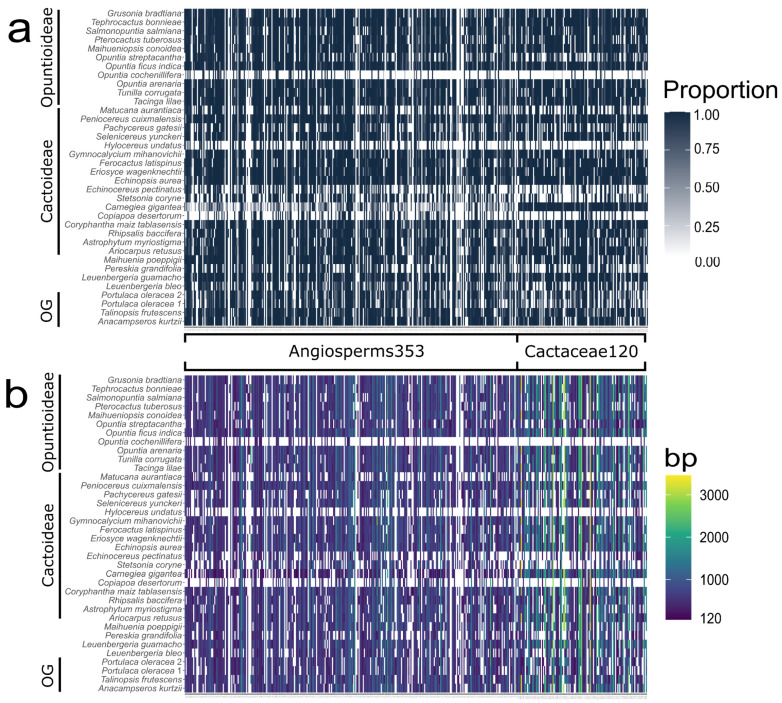
Heatmap plot showing recovery efficiency. Columns represent the targeted loci grouped based on probe sets and rows represent the 36 transcriptomes grouped based on the main groups in Cactaceae (OG: outgroup). (**a**) Length proportion of the target sequences recovered. (**b**) Size of the target gene sequences recovered.

Our analysis using MarkerMiner (MM) on available transcriptomes recovered 1859 mostly single-copy genes and 133 strictly single-copy genes. We decided to focus only on the strictly single-copy genes to avoid major issues with homology. Of these 133 strictly single copy loci, 8 were discarded due to not meeting the required parameters (see Methods Section). Additionally, five loci were discarded as a result of high BLAST matches to either the mitochondrial or chloroplast genomes and also to the Angiosperms353 probe sets. Our final *Cactaceae120* set consisted of 120 loci (Table S1 and SM1) represented by 469 exons, 740 reference sequences and a total of 136,495 bp sequence data (SM1). The reference sequence size varied between 123–3460 bp (Figure 2b and Figure S4b) with a mean of 1167 bp. The overall target capture performance for the 36 transcriptomes can be observed in Figure 2A showing a considerable amount (23.4%) of sequences with no hits. We also observed a wide range of values in the Hybpiper descriptive statistics (Figure 2a and Figure S2, Table S4). As expected, the numbers of reads varied widely as well as the reads mapped to our Cactaceae120 loci. The percentage of on-target reads varied between the groups (Figure S2a), but overall showed a very low mean value (0.12%). The rest of the reported descriptors, genes with contigs, genes with sequences and number of loci with ≥25%, ≥50%, or ≥75% target length, showed very similar patterns with mean values of 99, 93, 91, 88, and 84, respectively (Figure S2b–f). Additionally, we recovered 29 paralog warnings distributed across 13 samples; 15 of these warnings corresponded to outgroups. Finally, we found that the loci recovered differed between the main clades in Cactaceae (Table 2 and Table S3): from our Cactoideae samples, 120 loci were retrieved, followed by Opuntioideae (119 loci), outgroups (117 loci), *Leuenbergeria* + *Pereskia* (109 loci), and lastly, *Maihuenia* with 92 loci recovered. We also discovered that the highest locus overlap occurred between Opuntioideae and Cactoidae (119 loci) and the least overlap was between *Maihuenia* and *Leuenbergeria* + *Pereskia* (91 loci).

### 3.2. Angiosperms353

The 322 loci had a mean length of 702 bp, a size range of 120–2322 bp and 226,068 bp total sequence data, with 1 reference sequence per locus. Within the 322 loci selected for Cactaceae, 11 were not recovered in the overall 36 transcriptomes, leaving 311 loci for downstream analyses (Table S2). We observed a wide range of values in the Hybpiper descriptive statistics (Table S5 and Figure S3). As expected, the number of reads varied widely, as well as the reads mapped to our Angiosperms353 (A353) loci. The percentage of on-target reads differed between the groups (Figure S3A), but overall showed a low (mean 1.37%) value. Of the rest of the descriptors reported, genes with contigs, genes with sequences, and a number of loci with ≥25%, 50%, or 75% target length showed very similar patterns with mean values of 251, 240, 237, 226, and 209, respectively. Additionally, we recovered 234 paralog warnings distributed in 33 samples, with 59 of these warnings corresponding to outgroups (Table S5). Furthermore, we found differences in the loci recovered in the major clades across Cactaceae (Table 2): 303 loci were recovered for Cactoideae samples, followed by Opuntioideae (295 loci), outgroups (292 loci), *Leuenbergeria* + *Pereskia* (273 loci), and lastly *Maihuenia* with 254 loci. Additionally, we discovered that the highest overlap occurred between Opuntioideae and Cactoidae (288 loci) and Cactoideae and the outgroups (288 loci). On the other hand, the least overlap was between *Maihuenia* vs. *Leuenbergeria* + *Pereskia* (246 loci).

### 3.3. Phylogenetic Results

**Figure 3:**
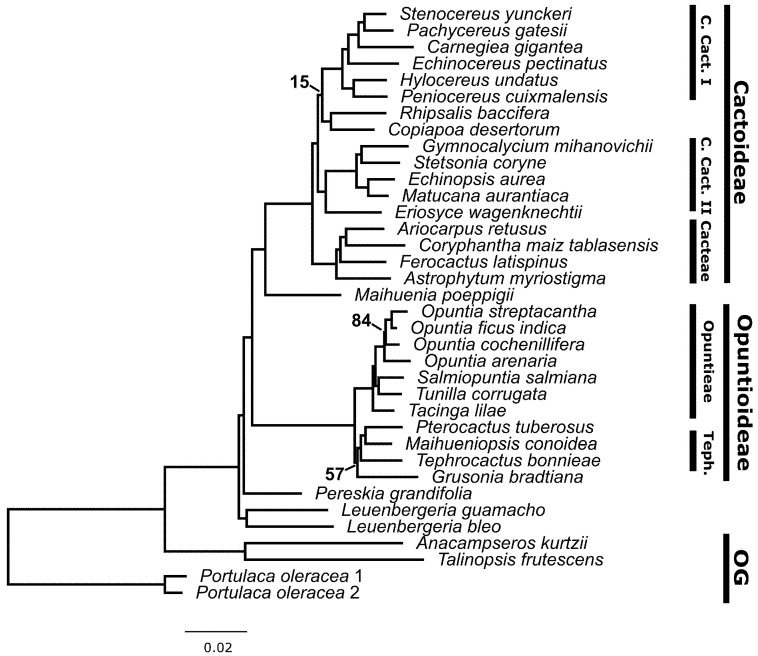
RAxML best-scoring ML tree of the concatenated database of the two probe sets. All nodes have ≥95% support value bootstrap unless noted. OG: Outgroups; Teph.: Tephrocacteae; C. Cact.: Core Cactoideae.

The combined RAxML phylogenetic reconstruction (Figure 3) showed overall very high support values, except for three relationships: (1) *Grusonia* sister to the rest of Opuntioideae (57% bootstrap support), (2) the clade containing *Opuntia cochenillifera* (84% bootstrap support) and (3) the *Rhipsalis* + *Copiapoa* relationship to the rest of core Cactoideae I (15% bootstrap support). This topology recovered *Leuenbergeria* as the sister clade to the rest of Cactaceae, followed by *Pereskia* s.s. We then recovered Opuntioideae as a monophyletic group; this clade included Opuntieae and Tephrocacteae + *Grusonia* (Cylindropuntieae). The sister to Opuntioideae was the *Maihuenia* + Cactoideae clade. Finally, within this group, we recovered Cacteae as a sister to core Cactoideae I + (*Rhipsalis* + *Copiapoa*) + Cactoideae II.

The separate RAxML analyses also recovered highly supported phylogenies (Figure S1). The Cactaceae120 probe set analysis (Figure S1a) recovered a similar topology to the combined dataset (Figure 3). This topology showed low support values only for the relationship of *Grusonia* to Opuntioideae (79% bootstrap support). On the other hand, the C120 probe set topology recovered *Leuenbergeria* and *Pereskia* as successive sister groups to the rest of Cactaceae and the *Rhipsalis* + *Copiapoa* clade as sister to the core Cactoideae I + core Cactoideae II clade (100% bootstrap support). Conversely, the A353 probe set analysis (Figure S1B) showed four nodes with low support: (1) *Leuenbergeria* as sister to the rest of Cactaceae except for *Pereskia* (73% bootstrap support), (2) *Grusonia* as sister to the rest of Opuntioideae (19% bootstrap support), (3) the node with *Opuntia cochenillifera* and relatives (73% bootstrap support), and (4) the node with *Echinocereus* and relatives (54% bootstrap support). This analysis recovered the *Copiapoa* + *Rhipsalis* clade as a sister group to the rest to core Cactoideae I (100% bootstrap support).

**Figure 4:**
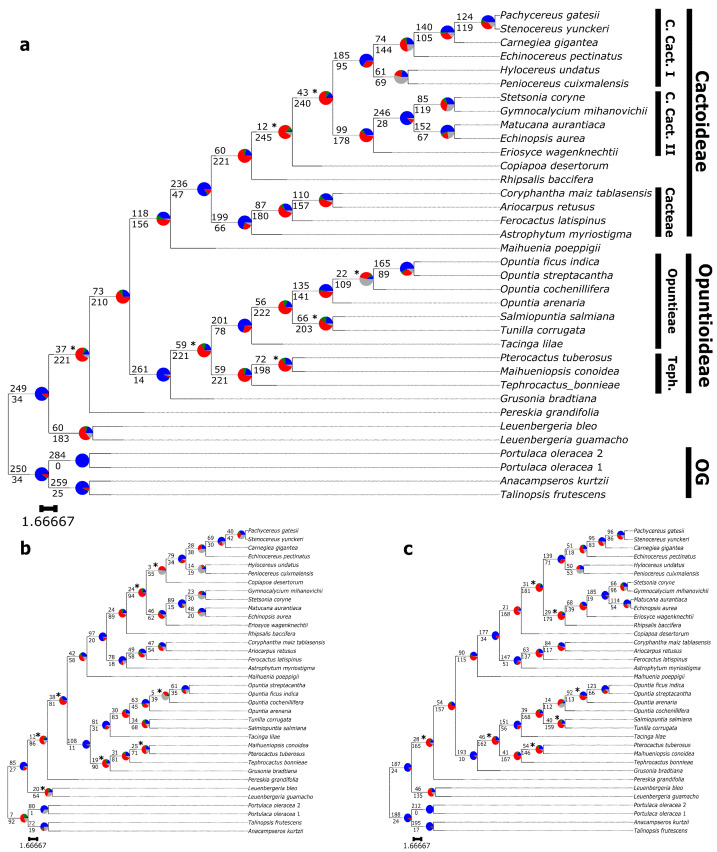
ASTRAL species tree with pie charts on the nodes representing gene tree topology proportions: blue represents concordance with the shown topology, green is the top alternative bipartition; red is all the other alternative bipartitions; and grey is no support for conflicting bipartition. Branch values represent concordance (top) and conflicting (bottom) gene trees. Asterisks mark all nodes with a posterior probability of ≤90 and scale bars represent coalescent units. (**a**) C120 and A353 probe sets combined. OG: Outgroups; Teph.: Tephrocacteae; C. Cact.: Core Cactoideae. (**b**) C120 probe set. (**c**) A353 probe set.

Our ASTRAL analyses showed overall high local posterior probability (LPP) values for the combined and separate datasets (Figure 4). Likewise, Phyparts results exhibited mostly a similar pattern in the combined and separate datasets. The combined dataset showed 7 of the 34 internal nodes with ≤90.0 LPP values (Figure 4a). The combined dataset (Figure 4a) species tree recovered Cactaceae and all the outgroups as well supported, with very few conflicting gene trees. *Leuenbergeria* was recovered as a sister to the rest of Cactaceae with little conflict. We then recovered *Pereskia* as sister to the rest of Cactaceae; this relationship was poorly supported (0.7 LPP) and showed ~85% conflicting gene trees. The Cactoideae + Opuntioideae node was well supported, but showed a high level of conflict (~74%). The Cactoideae + *Maihuenia* node was well supported and showed ~56% of conflicting gene trees. In contrast, the Cactoideae crown node was well supported and showed little conflict with the gene trees (16%). Within Cactoideae, Cacteae monophyly was well supported and showed little conflict (24%). We then recovered *Rhipsalis* (1 LPP) and *Copiapoa* (0.36 LPP) as successive sister lineages to core Cactoideae with considerable levels of conflict (≥70%). Core Cactoideae showed high conflict (84%), low support (0.54 LPP) and included considerable nodes with non-informative gene tree proportions. Core Cactoideae I was well supported with some conflict (33%), while core Cactoideae II displayed high support and conflict (64%). The major clade Opuntioideae was highly supported and showed little conflict (~5%), including *Grusonia* as sister to a Tephrocacteae + Opuntieae clade. The Tephrocacteae + Opuntieae node was poorly supported (~0.6 LPP) and had ~78% of gene trees conflicting with this relationship. Lastly, we observed highly uninformative (83%) gene nodes within *Opuntia*, also accompanied by low LPP (0.69).

Both separate C120 and A353 ASTRAL results (Figure 4b,c) displayed high support values with only eight and seven low-supported nodes, respectively. The Markerminer probe set topology showed a considerable amount of uninformative gene trees in 10 nodes (Figure 4b), while the Angiosperms353 probe set topology (Figure 4c) included 4 uninformative nodes. Additionally, the outgroups showed higher conflict in the C120 results than in the A353 tree. We found the Cactaceae crown node was well supported for both probe sets, and the A353 analysis showed the lowest conflict for this node. Next, *Leuenbergeria* was recovered as the sister to the rest of the Cactaceae in both trees, with similar patterns of gene trees but with C120 showing low support for this node (0.8 LPP). We then recovered *Pereskia* as the sister lineage to Cactoideae (including *Maihuenia*) + Opuntioideae with low support in both data sets (0.81 LPP C120 and 0.49 LPP A353) and the same pattern of a high proportion of conflicting gene trees. Next, the Cactoideae + Opuntioideae node displayed very similar patterns of high conflict; however, the C120 topology had low support (0.73 LPP) for this node. The Opuntioideae and Cactoideae clades were well supported and showed very little conflict in both C120 and A353 topologies. *Maihuenia* was recovered in both analyses as sister to Cactoideae with some conflict level and more uninformative trees in the C120 results. Within Cactoideae, Cacteae was recovered in both data sets with high support and similar proportions of concordant trees, although the C120 dataset included more noninformative gene trees for this node. We then observed that the relationships in Core Cactoideae showed differences between C120 and A353 ASTRAL results. The Core Cactoideae crown node was strongly supported in both analyses, also showing high levels of conflict, but it also included *Rhipsalis* (C120) or *Copiapoa* (A353) as sister lineages to the rest of Core Cactoideae. Next, Core Cactoideae I, including *Copiapoa,* was poorly supported (0.46 LPP) in the C120 topology and included more than 50% of uninformative gene trees. In contrast, the A353 Core Cactoideae I did not include *Copiapoa,* and its crown node showed high support and low conflict. Core Cactoideae II showed strong support in the C120 topology, but its crown node had considerable conflicting gene trees, while the A353 results recovered a weakly conflicting relationship (0.49 LPP) with *Rhipsalis* as the sister lineage to the rest of Core Cactoideae II. The Opuntioideae clade was recovered in both probe sets with high support and little conflict. In both trees the Opuntieae crown node was well supported, and it included some conflicting gene trees. Tephrocacteae was resolved as sister to *Grusonia* (Cylindropuntieae), which formed a clade sister to Opuntieae, when analyzing C120 data only, showing high levels of conflict and low support (0.54b LPP). In contrast, the A353 dataset showed *Grusonia* as sister to the rest of Opuntioideae.

### 3.4. Experimental Evaluation

Our experimental test exhibited different recovery patterns for the two probe sets (Figure S4a). The A353 set showed an irregular pattern (29% no hits) of the sequence recovery with no apparent difference between the groups. A total of 28 loci showed ≤3 hits and 10 of the 11 loci absent in our transcriptome analysis (see Section 3.2) were also absent in our A353 experimental dataset. In addition, only 5% of the sequences recovered were ≥1000 bp long (Figure S4b), while 47% of hits corresponded to short fragments (≤500 bp). In contrast, the C120 probe set showed a very consistent pattern with less than 3% of missing hits. The size of fragments varied widely with 45% of sequences being ≥1000 bp long and 28% having ≤500 bp fragments.

## 4. Discussion

The phylogenetic results based on the separate C120 and A353 datasets, as well as the concatenated datasets of both, are in line with previous phylogenetic hypotheses regarding incongruence, as well as well-supported topologies. *Leuenbergeria* was recovered as a sister to *Pereskia* + the rest of Cactaceae, as in Edwards et al. [38] and Walker et al. [39], thereby reaffirming the paraphyly of the traditional “*Pereskia* s.l.”. Within Opuntioideae, two conflicting topologies were recovered, one with Cylindropuntieae + Tephrocacteae as a sister to Opuntieae and the other with Cylindropuntieae as a sister to a Tephrocactaceae + Opuntieae clade. Plastome data revealed the topological scenario with Opuntieae as a sister to a Cylindropuntieae + Tephrocacteae clade [11], while transcriptome data revealed the latter scenario, with Cylindropuntieae as a sister to an Opuntieae + Tephrocacteae clade [39]. Both of these scenarios are poorly supported in this paper (between 19–79% bs; Figure 1, 0.54–0.6 LPP; Figure 4 and Figure S1a,b), and we confirmed a high degree of conflict around that node based on transcriptome data. Plastome data, on the other hand, resolve the relationship with Opuntieae as a sister to Tephrocacteae + Cylindropuntieae with high support [11,41]. Based on our results, it seems likely that using supercontigs, not just exons, as well as increasing taxon sampling of Cylindropuntieae may provide further support for these deep relationships, and we are currently testing this hypothesis with our more comprehensive dataset of these groups. Although high numbers of polyploids are well documented from all three tribes in Opuntioideae [51], there seems to be no reason to suspect that allopolyploidization is the reason for the congruence seen here, given that diploids also are common throughout those three clades. Thus, diploidy certainly is the ancestral state for Opuntioideae.

Within Cactoideae, *Rhipsalis* and *Copiapoa* were recovered as either a sister clade to Core Cactoideae I or Core Cactoideae II (RAxML), with high support for both scenarios. In contrast, ASTRAL did not recover these relationships in either the combined or separate datasets, and it showed conflicting results between the three datasets. This is not surprising given that both of these clades are isolated lineages [8], and previous phylogenetic topologies based on transcriptome data and poor taxon sampling have shown conflicting topological signals around the *Copiapoa* node. Increasing taxon sampling likely will ameliorate this topological issue. Notably, the isolated lineage *Calymmanthium*, which has been shown to be sister to Core Cactoideae I and II, was not sampled in this study and would likely provide topological stability in this part of the tree (eight and Majure et al. (unpubl. data)).

We compared our gene-tree conflict analyses with Wang et al. [52] and discovered most of the main clades in Cactaceae were recovered in both studies with similar proportions of concordance. In contrast, our results showed more conflicting and less uninformative gene tree proportions for the rest of the relationships within the family. Likewise, we noticed that higher levels of uninformative nodes were present in the Markerminer dataset when compared to the Angiosperms353 dataset, similar to patterns found in Asteraceae [22] and contrary to Malinae (Rosaceae) [21]. This discovery was unexpected because we predicted that a Cactaceae-specific probe set would help to solve the most conflicting relationships within the family similar to Gesneriaceae in [23] and Malinae in Ufimov et al. [21]. On the contrary, based on these preliminary results using 36 transcriptomes, it appears the A353 probe set is more informative than the Cactaceae-specific probe set. Nevertheless, again we predict these patterns will change with new sequencing and the inclusion of supercontigs (similar to [25]).

Several of the unresolved nodes both in RAxML and in ASTRAL analyses coincided with samples with low target recovery (e.g., *O. cochenillifera* and *C. desertorum*). Although most problematic nodes coincided with low reads mapped to our target genes, this was not an indicator of the quality of the original sequences (Tables S4 and S5). A more comprehensive sampling across these lineages and Cactaceae is necessary to confirm if they definitively lack the target regions we used. In a similar way, we expected the number of sequences mapped to vary between clades and probe sets. We only recovered differences between the percentage of read of targets, where the C120 dataset showed less variation than the A353 dataset (Figures S2 and S3). In contrast, we observed differences between the genes recovered for each clade (Table 2), with Cactoideae showing the higher recovery in both sets. We expect both metrics to maintain these patterns for Cactoideae and Opuntioideae, as they were the two most sampled clades in this study.

Overall, the Cactaceae120 probe set showed a higher recovery efficiency in the experimental evaluation compared to the transcriptome evaluation. In contrast, the Angiosperms353 probe set performed suboptimal in the experimental test compared to C120 and to the transcriptome results. These results coincide with several studies that include group specific probe sets that outperform Angiosperms353 [22,24]. However, the pattern on our A353 results could be a product of including only one reference sequence per locus [53]. This outcome could be potentially improved using the mega353 pipeline [54], as this pipeline uses more reference sequences per target to call the loci [55]. Nevertheless, our results are a strong foundation for future applications (similar to [21]) of the Angiosperms353 probe set focused on such a diverse group, such as Cactaceae.

Finally, our probe set derived from transcriptome data and incorporating phylogenetically informative probes from A353 for Cactaceae yielded a 431-locus probe set capable of reconstructing relationships among and within the major clades of Cactaceae with mostly high support. Increased taxon sampling and the use of supercontigs, rather than just exonic regions, will most surely clarify topological inconsistencies recovered with the current dataset. As a consequence, we are currently working on a phylogenomic study based on the experimental dataset mentioned here. Phylogenomic comparisons with our probe set data and plastome datasets have the potential to reveal biologically important patterns, which have led to the generation of the considerable macromorphological, physiological and anatomical diversity exhibited by the Cactaceae across the Americas.

## Figures and Tables

**Table 1:** Transcriptomes used for phylogenetic reconstruction in this study.

Species	Clade	NCBI SRA #
*Anacampseros kurtzii*	Outgroup	SRR6435355 (Bioproject: PRJNA428216)
*Ariocarpus retusus*	Cactoideae	SRR7905834
*Astrophytum myriostigma*	Cactoideae	SRR7905836
*Carnegiea gigantea*	Cactoideae	SRR5036296
*Copiapoa desertorum*	Cactoideae	SRR7905838 (Bioproject: PRJNA493215)
*Coryphantha maiz tablasensis*	Cactoideae	SRR7905839 (Bioproject: PRJNA493215)
*Echinocereus pectinatus*	Cactoideae	SRR1698109 (Bioproject: PRJNA269655)
*Echinopsis aurea*	Cactoideae	SRR7905840 (Bioproject: PRJNA493215)
*Eriosyce wagenknechtii*	Cactoideae	SRR7905831 (Bioproject: PRJNA493215)
*Ferocactus latispinus*	Cactoideae	SRR7905830 (Bioproject: PRJNA493215)
*Grusonia bradtiana*	Opuntioideae	SRR7905852 (Bioproject: PRJNA493215)
*Gymnocalycium mihanovichii*	Cactoideae	SRR7905853 (Bioproject: PRJNA493215)
*Hylocereus undatus*	Cactoideae	SRR11603181
*Leuenbergeria bleo*	*Leuenbergeria* + *Pereskia*	SRR1698112
*Leuenbergeria guamacho*	*Leuenbergeria* + *Pereskia*	SRR7905854 (Bioproject: PRJNA493215)
*Maihuenia poeppigii*	*Maihuenia*	SRR7905849 (Bioproject: PRJNA493215)
*Maihueniopsis conoidea*	Opuntioideae	SRR7905848 (Bioproject: PRJNA493215)
*Matucana aurantiaca*	Cactoideae	SRR7905855 (Bioproject: PRJNA493215)
*Opuntia arenaria*	Opuntioideae	SRR7905850 (Bioproject: PRJNA493215)
*O. cochenillifera*	Opuntioideae	SRR1698108
*O. ficus indica*	Opuntioideae	SRR3567682
*O. streptacantha*	Opuntioideae	SRR3478181
*Pachycereus gatesii*	Cactoideae	SRR7905847 (Bioproject: PRJNA493215)
*Peniocereus cuixmalensis*	Cactoideae	SRR7905861 (Bioproject: PRJNA493215)
*Pereskia grandifolia*	*Leuenbergeria* + *Pereskia*	SRR1698106
*Portulaca oleracea* 1	Outgroup	SRR10247085
*P. oleracea* 2	Outgroup	SRR10247116
*Pterocactus tuberosus*	Opuntioideae	SRR7905860 (Bioproject: PRJNA493215)
*Rhipsalis baccifera*	Cactoideae	SRR7905851 (Bioproject: PRJNA493215)
*Salmiopuntia salmiana*	Opuntioideae	SRR7905862 (Bioproject: PRJNA493215)
*Stenocereus yunckeri*	Cactoideae	SRR7905856 (Bioproject: PRJNA493215)
*Stetsonia coryne*	Cactoideae	SRR7905865(Bioproject: PRJNA493215)
*Tacinga lilae*	Opuntioideae	SRR7905864 (Bioproject: PRJNA493215)
*Talinopsis frutescens*	Outgroup	SRR6435354 (Bioproject: PRJNA428216)
*Tephrocactus bonnieae*	Opuntioideae	SRR7905863 (Bioproject: PRJNA493215)
*Tunilla corrugata*	Opuntioideae	SRR7905866 (Bioproject: PRJNA493215)

**Table 2:** Comparison of loci recovered per probe set and main group in Cactaceae. Numbers in white cells represent the number of loci shared between main groups in Angiosperms353 (above the diagonal line) and Cactaceae120 (below the diagonal line). The gray diagonal cells correspond to the number of loci recovered per probe set in each of the main groups.

		Angiosperms353				
		Cactoideae	*Leuenbergeria* + *Pereskia*	*Maihuenia*	Opuntioideae	Outgroup
Cactaceae120	Cactoideae	303 120	272	252	288	288
	*Leuenbergeria* + *Pereskia*	109	273 109	246	267	269
	*Maihuenia*	92	91	254 92	249	253
	Opuntioideae	119	109	92	295 119	281
	Outgroup	117	108	92	116	292 117

## Data Availability

Both probe sets used in this study are available on Dryad data repository (doi:10.5061/dryad.k3j9kd58k; https://datadryad.org/stash/share/oEistPRiyNCE4gHDC0lIuHzVM5QXriAtBUp7_wvHDcM accessed on 1 February 2022).

## Supplementary Materials

The following are available online at https://www.mdpi.com/article/10.3390/genes13020350/s1: Figure S1: RAxML best-scoring ML tree, all nodes have ≥95% support value bootstrap unless noted. (a) C120 probe set, (b) A353 probe set. Figure S2: Boxplots representing Cactaceae120 stats data generated by Hybpiper. Each boxplot represents a data group: Comb. (all the accessions combined), Cact. (Cactoideae), Leu + Per (*Leuenbergeria* and *Pereskia*), Mai. (*Maihuenia*), Opu. (Opuntioideae) and Out. (Outgroup). (a) Percentage of reads on target compared to the original reads, (b) Genes with contigs, (c) Genes with sequences, (d) Number of loci with ≥25% target length, (e) Number of loci with ≥50% target length, (f) Number of loci with ≥75% target length. Figure S3: Boxplots representing Angiosperms353 stats data generated by Hybpiper. Each boxplot represents a data group: Comb. (all the accessions combined), Cact. (Cactoideae), Leu + Per (*Leuenbergeria* and *Pereskia*), Mai. (*Maihuenia*), Opu. (Opuntioideae) and Out. (Outgroup). (a) Percentage of reads on target compared to the original reads, (b) Genes with contigs, (c) Genes with sequences, (d) Number of loci with ≥25% target length, (e) Number of loci with ≥50% target length, (f) Number of loci with ≥75% target length. Figure S4: Heatmap plot showing recovery efficiency. Columns represent the targeted loci grouped based on probe sets and rows represent the 96 samples sequenced in 4 groups: Opuntioideae, Cactoideae, *Leuenbergeria* + *Pereskia* (LP) and the Outgroups (O). (a) Length proportion of the target sequences recovered. (b) Size of the target sequences recovered. Table S1: MarkerMiner results using 15 transcriptomes as references. Table S2: Hybpiper target recovery of Angiosperms353 probe set with 36 transcriptomes. Table S3: Hybpiper target recovery of Cactaceae120 probe set with 36 transcriptomes. Table S4: Hybpiper statistics of Cactaceae120 with the 36 transcriptomes. Table S5: Hybpiper statistics of Angiosperms353 with the 36 transcriptomes. Table S6: Species list included in the experimental evaluation. SM1: Cactaceae120 and Angiosperms353 sequences used in this study (https://datadryad.org/stash/share/oEistPRiyNCE4gHDC0lIuHzVM5QXriAtBUp7_wvHDcM accessed on 1 February 2022).
