# Supplementary figures and images for: A New Approach Using Targeted Sequence Capture for Phylogenomic Studies across Cactaceae
Source: Genes (Basel). 2022 Feb 15;13(2):350. doi: 10.3390/genes13020350 (PMC8871817; doi:10.3390/genes13020350)

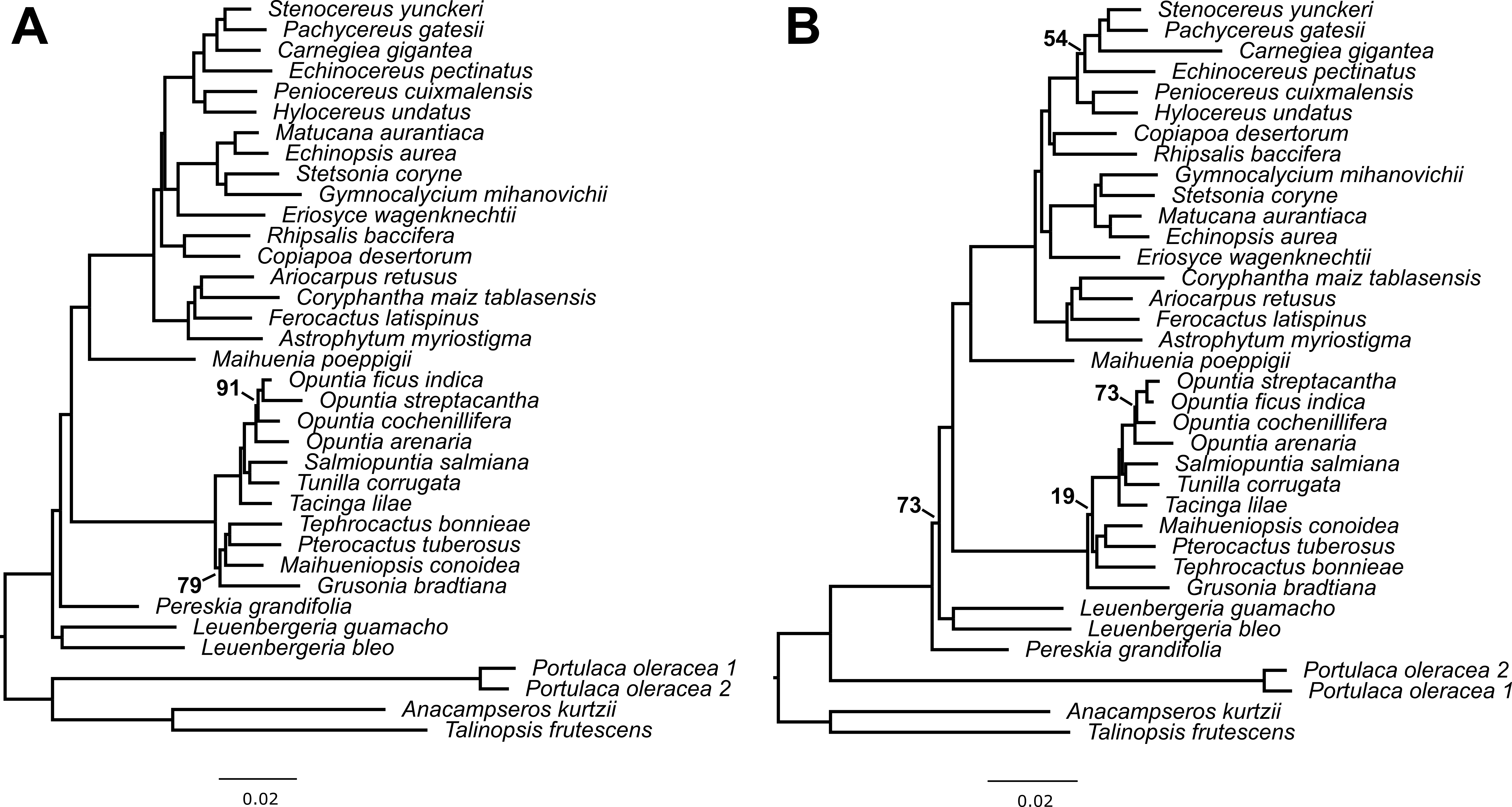

Supplement: Supplementary file 1 [file genes-13-00350-s001.zip › Figure S1.png]

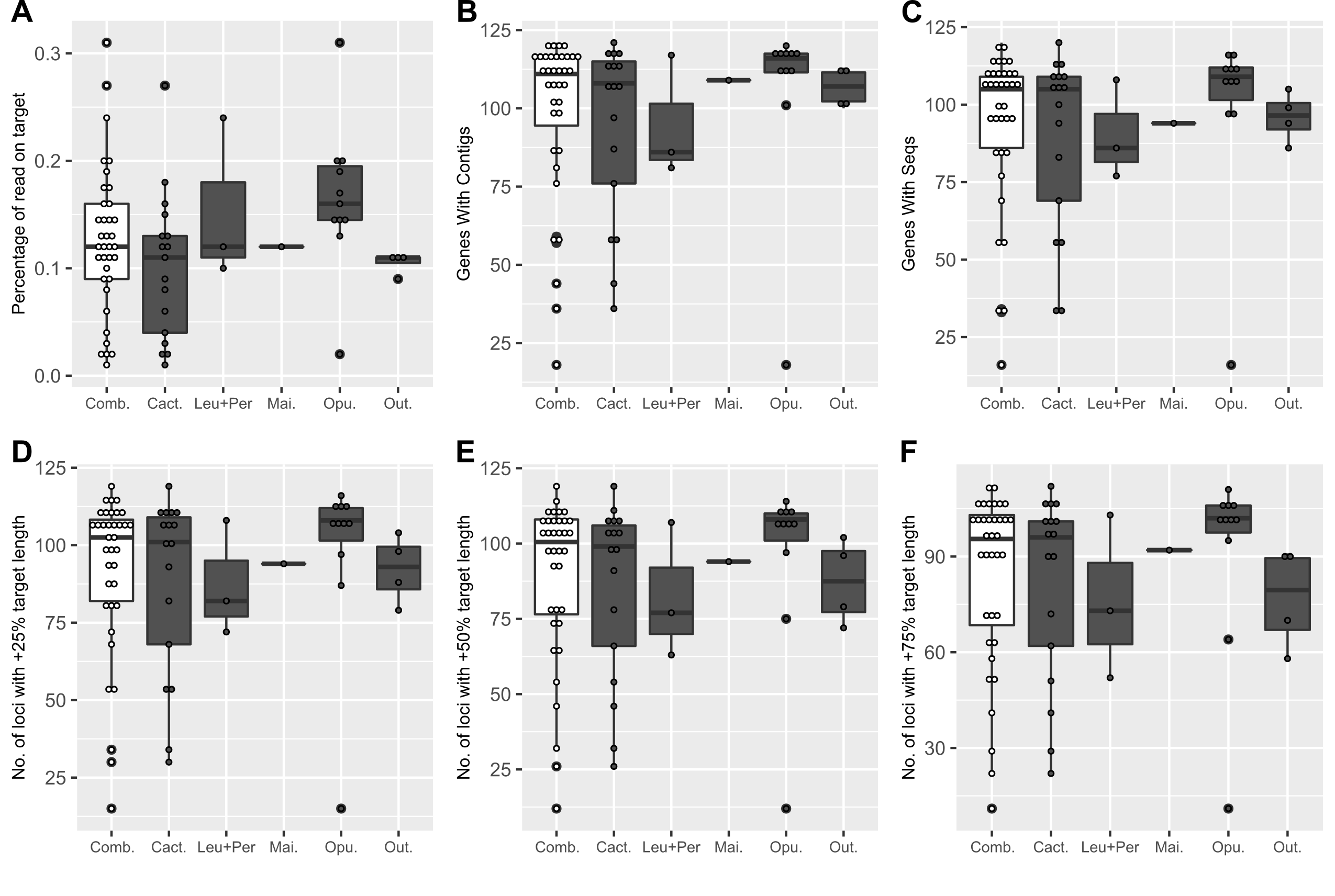

Supplement: Supplementary file 1 [file genes-13-00350-s001.zip › Figure S2.png]

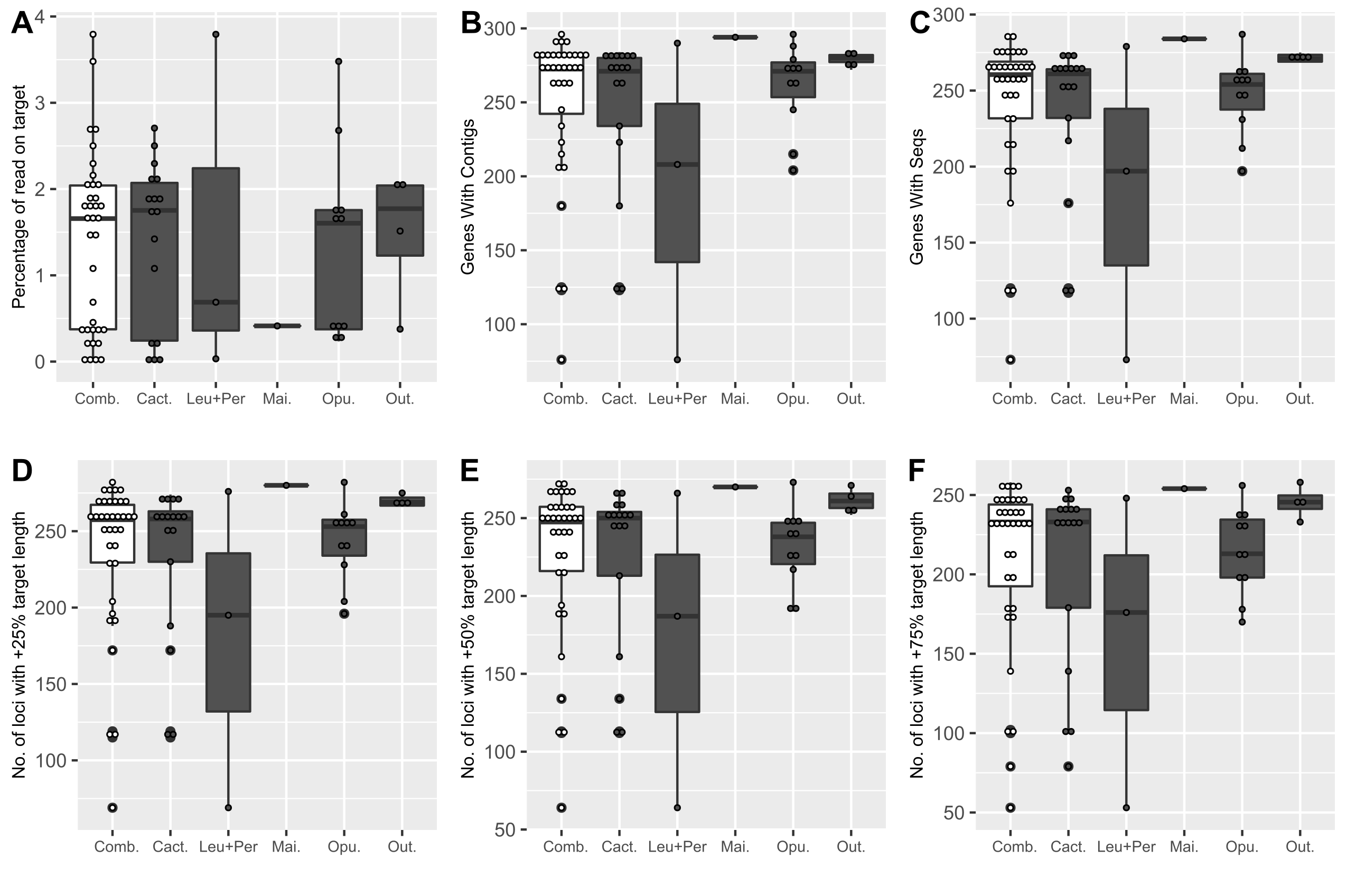

Supplement: Supplementary file 1 [file genes-13-00350-s001.zip › Figure S3.png]
